# A Dioxobilane as Product of a Divergent Path of Chlorophyll Breakdown in Norway Maple**

**DOI:** 10.1002/anie.201103934

**Published:** 2011-09-16

**Authors:** Thomas Müller, Martina Rafelsberger, Clemens Vergeiner, Bernhard Kräutler

**Affiliations:** Institute of Organic Chemistry and Center of Molecular Biosciences, University of InnsbruckInnrain 52a, 6020 Innsbruck (Austria)

**Keywords:** catabolites, chlorophyll, dyes/pigments, natural products, porphyrinoids

Chlorophyll breakdown is a hallmark of leaf senescence and a major contributor to the emergence of the fall colors.[1, 2] Strikingly, essential pieces of the puzzle of this biological phenomenon have been solved only within the last two decades.[2–5] A breakthrough was the identification and structure elucidation of a colorless tetrapyrrolic chlorophyll catabolite, thereafter named *Hv*-NCC-1.[6] The further characterization of tetrapyrrolic chlorophyll catabolites in higher plants has meanwhile provided the structural basis for detailed insight into the pathway of chlorophyll breakdown.[7, 8] Colorless tetrapyrroles, such as *Hv*-NCC-1, typically accumulate in senescent leaves of higher plants and they have been classified as “nonfluorescent” chlorophyll catabolites (NCCs).[7] Indeed, NCCs have been considered to be the “final” breakdown products of a largely common and well-controlled “linear” catabolic pathway in senescent leaves (see Scheme 1).[7, 9]

**Scheme 1:**
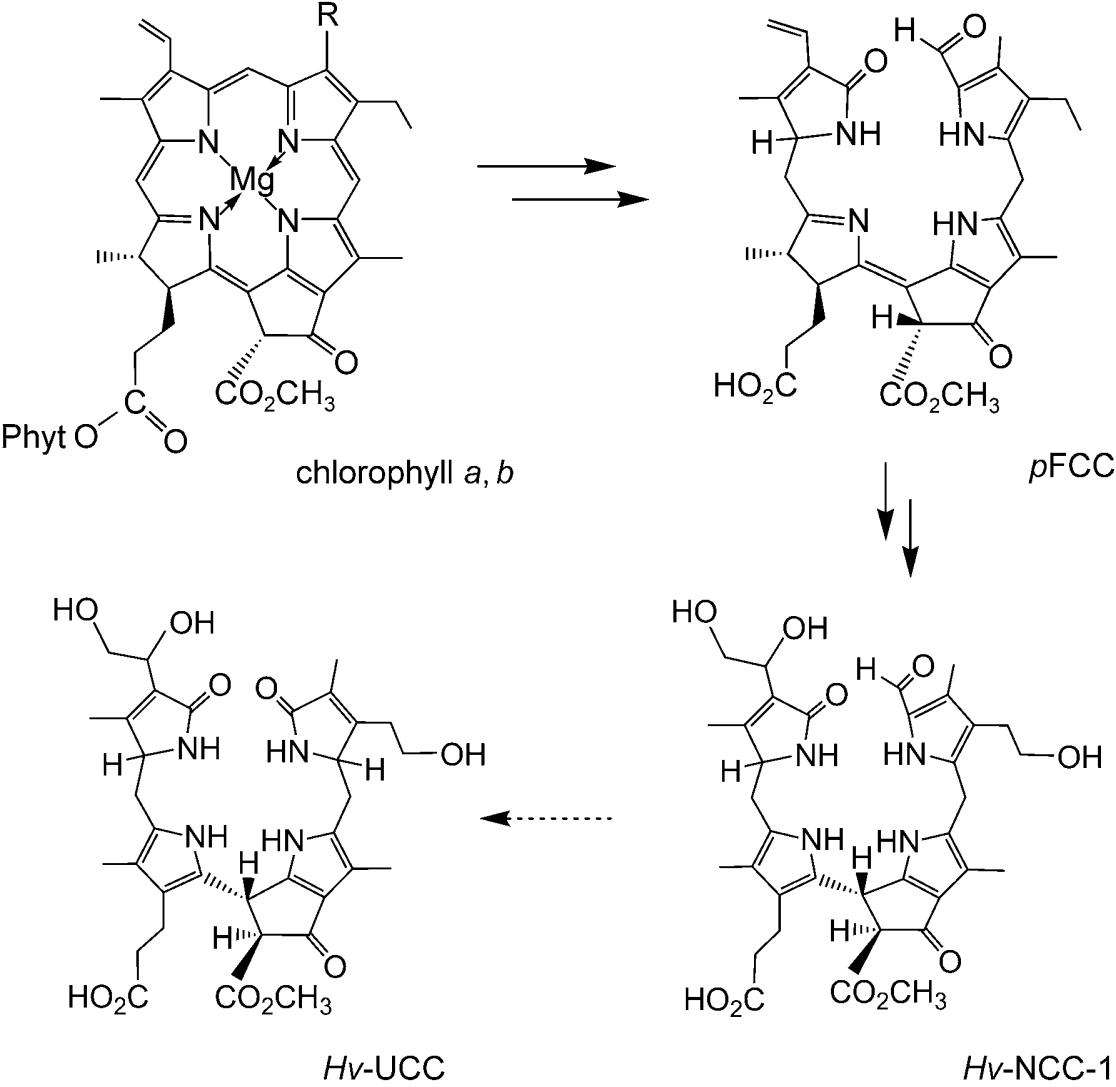
Short outline of chlorophyll breakdown represented by the structures of catabolites identified in barley (*Hordeum vulgare*).[6, 7] Chlorophyll *a* (R=CH_3_) and *b* (R=CH=O) are broken down in senescent chloroplasts to the primary FCC (*p*FCC). *p*FCC is modified by enzymes in the cytosol and the resulting *m*FCCs are exported into the vacuole, where NCCs (such as *Hv*-NCC-1) are formed by chemical isomerization. *Hv*-UCCs were detected in barley leaves and were suggested to be oxidation products of *Hv*-NCC-1.[10]

Here we describe studies of chlorophyll breakdown in senescent leaves of Norway maple (*Acer platanoídes*, see Figures S1 and S2 in the Supporting Information), a deciduous tree native to Eurasia.[11] Surprisingly, in fresh extracts from slightly degreened maple leaves no compounds with the absorption properties of the typical colorless chlorophyll catabolites could be detected by high-pressure liquid chromatography (HPLC). Extracts from further degreened leaves showed a similar analytical profile. In particular, no traces of the NCCs were found in any such senescent leaves. Instead one major compound was found that showed a UV absorption spectrum with broad bands near 237 nm and 274 nm (see Figures 1 and 2). The same spectrum was reported for two “urobilinogenoidic” chlorophyll catabolites,[10] which were found (besides *Hv*-NCC-1)[6] in senescent leaves of barley (*Hordeum vulgare*) and which were suggested to be generated by oxidation of *Hv*-NCC-1. Secondary oxidative transformations of NCCs have also been observed recently in other senescent leaves, in which yellow- and red-colored dehydrogenation products of the tetrapyrrolic NCC were identified.[12, 13]

**Figure 1:**
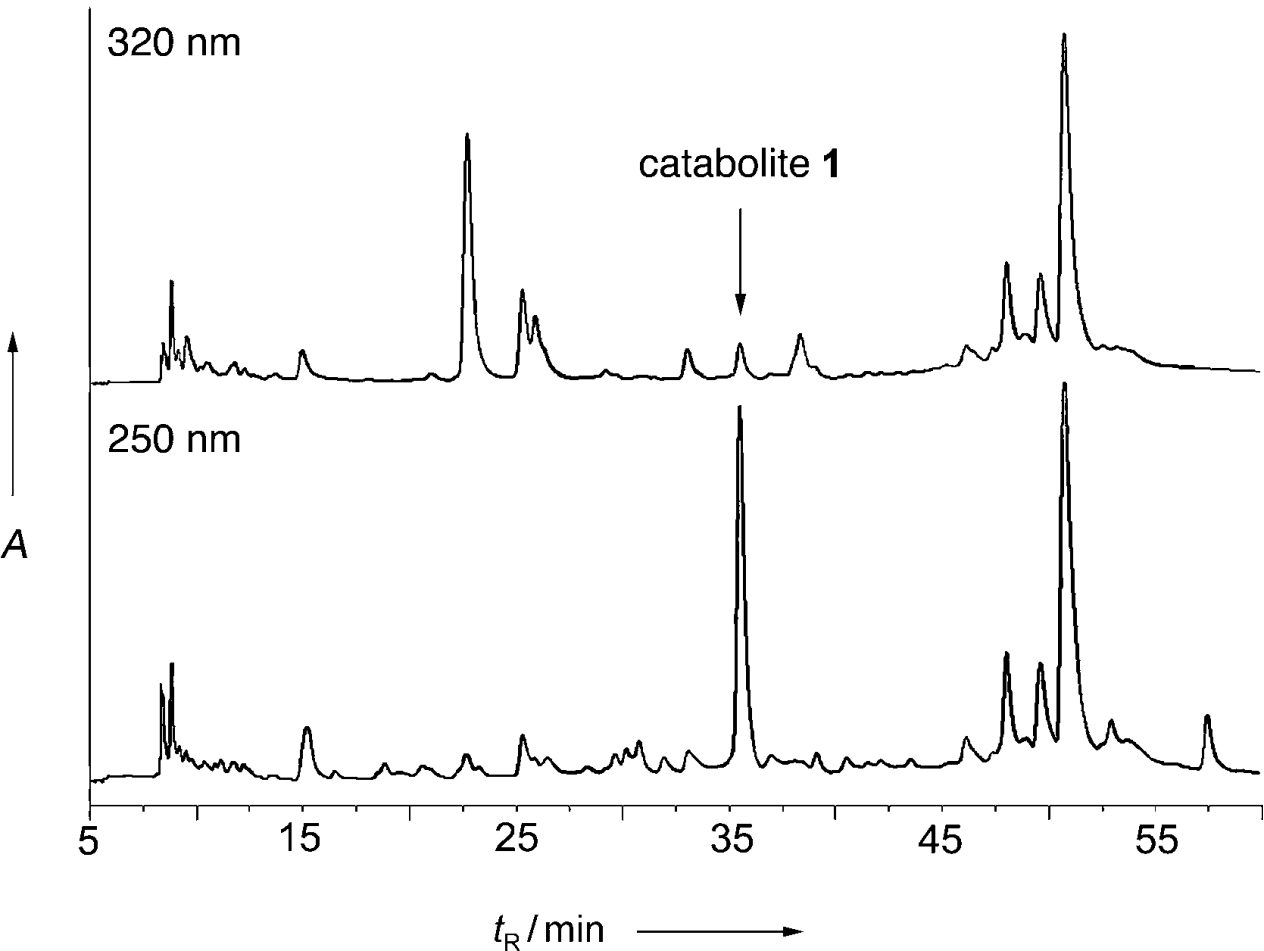
HPLC analysis of an extract of senescent leaves of Norway maple. The catabolite **1** gives rise to a strong signal at 250 nm and a weak one at 320 nm (where NCCs have a characteristic absorption).[8]

**Figure 2:**
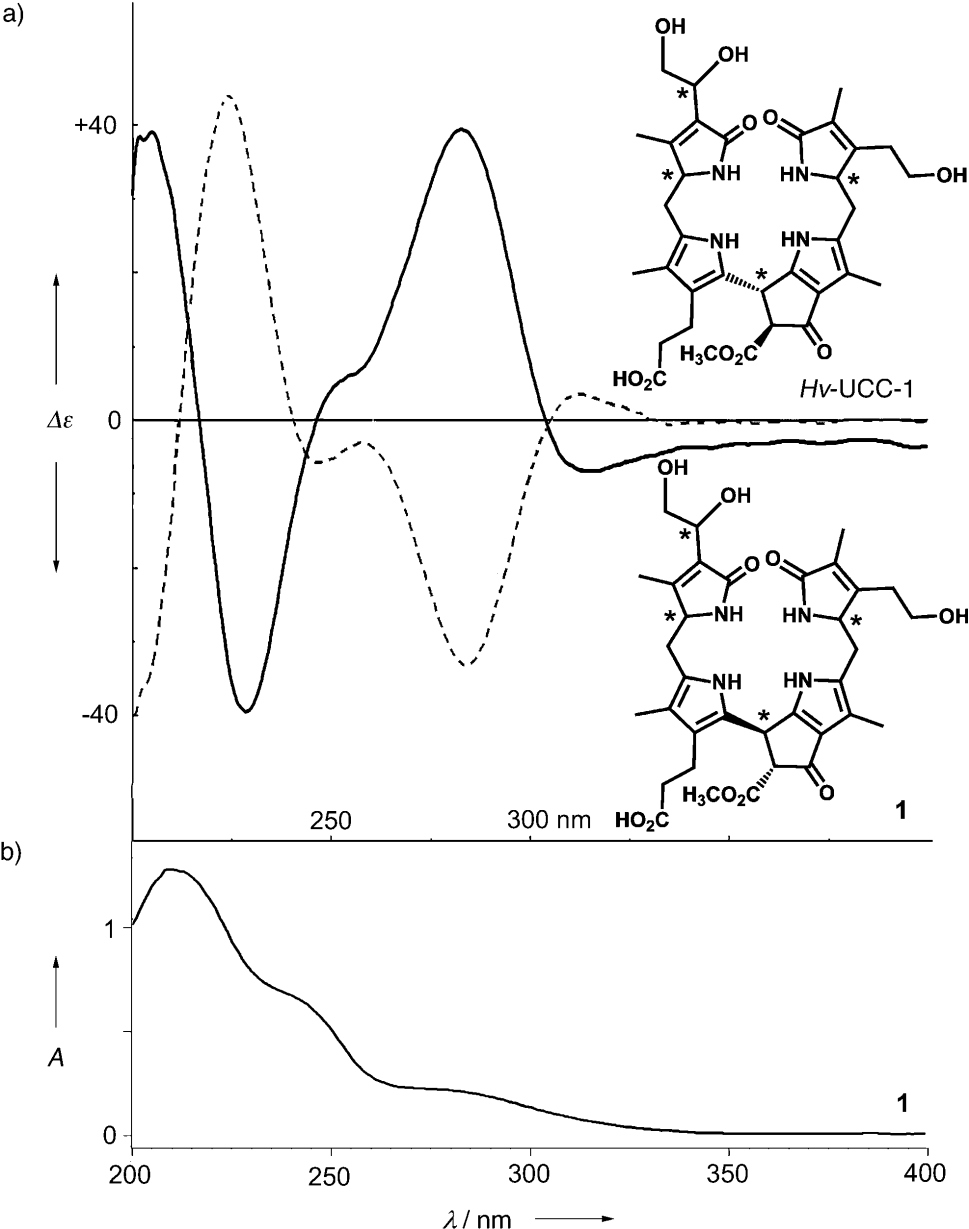
CD and UV spectra of the catabolite **1.** a) CD spectra of **1** (full line) and of *Hv*-UCC-1 (dashed line) in MeOH; b) UV absorption spectrum of **1** in MeOH.

An extract obtained from 130 g (wet weight) of leaves of Norway maple was purified first by medium-pressure liquid chromatography (MPLC), and an 88 mg raw sample of the catabolite **1** was obtained. A 20 mg portion of raw **1** was purified by preparative HPLC and dioxobilane **1** (4.8 mg) was isolated as a pale yellow powder. In the ESI mass spectrum[14] of **1** a prominent signal is observed at *m*/*z*=667.19 (due to [*M*+H]^+^, see below). A high-resolution ESI mass spectrum exhibits a strong signal at *m*/*z*=705.2532 corresponding to the pseudo-molecular ion [C_34_H_42_N_4_O_10_K]^+^ (*m*/*z*_calc_=705.2533) and confirming the molecular formula of C_34_H_42_N_4_O_10_ that was suggested for **1** from the ESI mass spectrum. The molecular constitution of **1** could be determined by multidimensional, homo- and heteronuclear NMR spectroscopy.[15] The ^1^H NMR spectrum of **1** (in CD_3_OD) shows signals for 33 hydrogen atoms. Four singlets of methyl groups stand out at high field; a fifth one, at *δ*=3.75 ppm, was assigned to a methyl ester group (see Figure S5 in the Supporting Information). However, there are no signals at low field, that is, at *δ*>5 ppm, which excludes the presence of vinyl and formyl groups in **1**. The molecular constitution was determined unambiguously from a set of ^1^H,^1^H-ROESY and ^1^H,^1^H-COSY experiments, as well as from ^1^H,^13^C-HSQC and ^1^H,^13^C-HMBC spectra, which allowed the assignment of all 33 non-exchangeable H atoms and of all C atoms (see Figures S3 and S4 in the Supporting Information). The constitution of the catabolite **1** was thus deduced to be that of a dioxobilane[16] (Scheme 2).

**Scheme 2:**
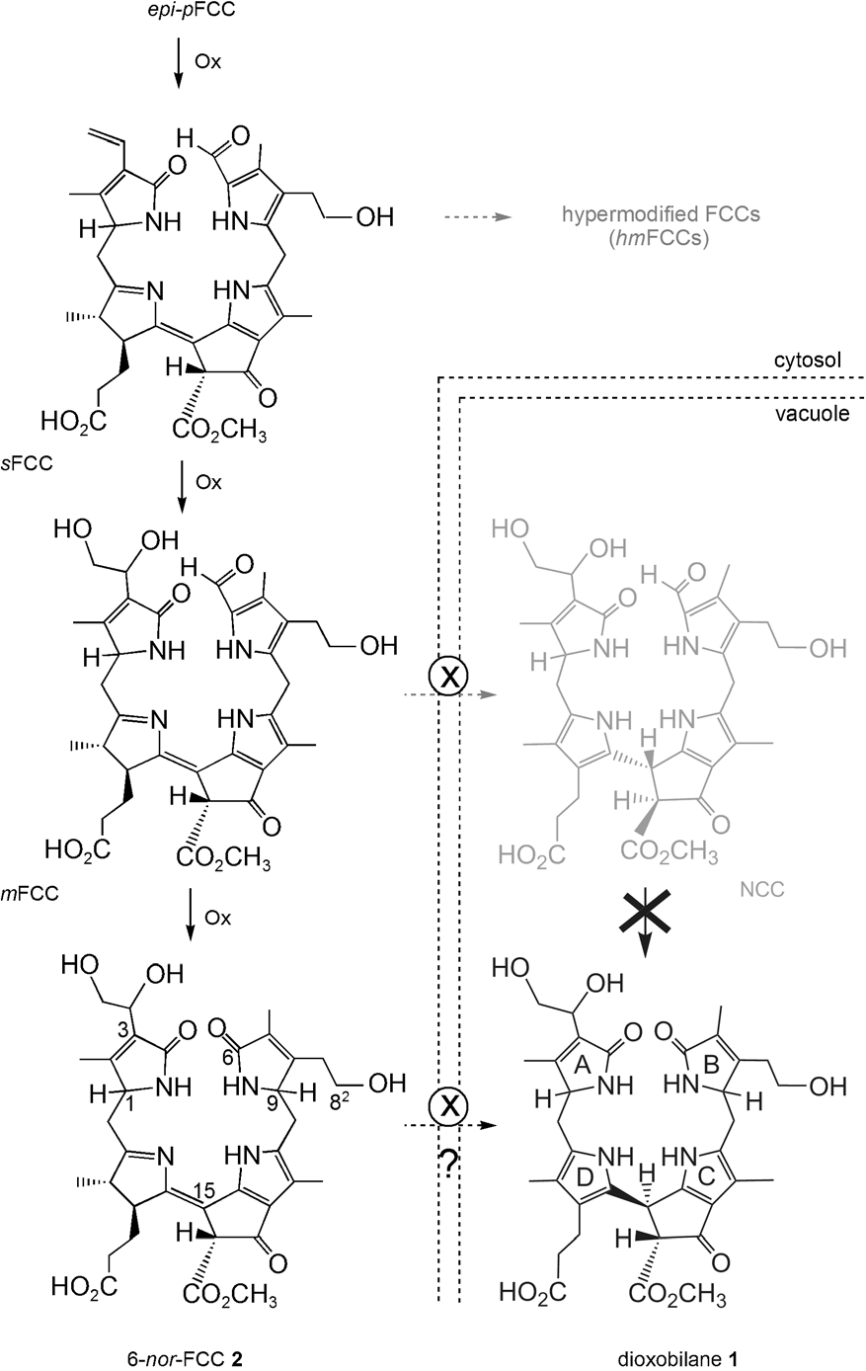
Structural outline of the proposed later part of chlorophyll in senescent leaves of Norway maple (*Acer platanoides*), which begins with a “primary” FCC, deduced to be *epi*-*p*FCC (the C-1 epimer of *p*FCC).[25] Hydroxylation at C-8^2^, followed (in an unknown sequence) by dihydroxylation of the vinyl group at C-3 and oxidative deformylation at C-6, probably furnishes the still-elusive 6-*nor*-FCC **2** in the cytosol. The catabolite **1** may be formed in the vacuole by a hypothetical isomerization of **2** and transport of **2** into the vacuole by the catabolite transporter X. Hypermodified FCCs (*hm*FCCs, found in bananas)[29] and NCCs, as found in senescent leaves of barley[6], were not detected in the leaves of Norway maple.

Thus the constitution of dioxobilane **1** turns out to be identical to that of the two epimeric “urobilinogenoidic” chlorophyll catabolites identified in senescent leaves of barley, and which are named *Hv*-UCC-1 and *Hv*-UCC-2 here.[10] In HPLC co-injection dioxobilane **1** and *Hv*-UCC-1 (the more polar and major “urogenobilinoidic” catabolite from senescent barley leaves) also displayed the same retention characteristics (see Figure S6 in the Supporting Information). However, we discovered a strikingly contrasting spectroscopic feature of **1** when we compared its circular dichroism (CD) spectrum with that of *Hv*-UCC-1: the CD spectrum of **1** is practically a mirror image of the spectra of *Hv*-UCC-1 (see Figure 2) and its (presumed) C-9 epimer.[10, 17]

Interestingly, the CD spectra of the two epimeric *Hv*-UCCs (*Hv*-UCC-1 and *Hv*-UCC-2) are similar,[10] showing the relative configuration at C-1 and C-9 to have only a minor influence on these spectra. In contrast, the near mirror symmetry in the CD spectra of **1** and of *Hv*-UCC-1 signifies a CD-relevant stereochemical difference. This difference is thus indicated to result from an opposite configuration at the C-15 *meso* position, between the (substituted) pyrrole rings C and D. This conclusion is consistent with the assignment of the broad absorption bands at longest wavelengths (i.e. at 274 nm and 238 nm) to these two rings[18] in the spectra of **1** and of the *Hv*-UCCs. Furthermore, the closely matching NMR and HPLC data of dioxobilane **1** and the isomeric *Hv*-UCC-1 also indicate their mutual structural similarity to include common relative configurations at C-15 and at C-13^2^, as well as at the two further stereocenters C-1 and C-9 of the tetrapyrrole skeleton (see Table SI in the Supporting Information). The thus-derived opposite configurations of C-1 in **1** and in *Hv*-UCC-1 are, in fact, compatible with the deduced existence of primary FCCs in barley and in *Acer* sp. that are the mutual (C-1) epimers (*p*FCC and *epi*-*p*FCC; FCC=fluorescent chlorophyll catabolite).[19] Further scrutiny by comparison of chemical shift and coupling values in the ^1^H NMR spectra of the catabolite **1** and (those published) of *Hv*-UCC-1[10] revealed the protons at or near the dihydroxyethyl substituent at C-3 to behave differently (see Table S1 in the Supporting Information). Such an NMR-detected difference suggests a different relative configuration at C-3^1^ with respect to C-1. All our findings characterize the dioxobilane **1** as the pseudo enantiomer of *Hv*-UCC-1 in which all stereogenic carbon centers, other than at the side-chain position C-3^1^, have the opposite absolute configuration (i.e. the same relative configuration) to those in *Hv*-UCC-1.

The deduced opposite configuration at C-15 in **1** and in *Hv*-UCC-1 is very intriguing: Indeed, C-15, the *meso* carbon between two pyrrole units, is resistant to epimerization not only in **1** and the *Hv*-UCCs,[10] but also in NCCs, such as *Hv*-NCC-1.[20] The “urogenobilinoidic” catabolites *Hv*-UCC-1 and *Hv*-UCC-2 have thus been suggested to have the same absolute configuration at C-15 (and at C-1) as their presumed precursor, *Hv*-NCC-1 (see Scheme 1).[10] All natural NCCs, in turn, have been proposed to exhibit the same configuration at the C-15 position.[20] During chlorophyll breakdown this saturated carbon center is generated in a stereospecific non-enzymatic isomerization of FCCs[21] that was deduced to yield NCCs with a common *R* configuration at C-15.[20, 22]

Indeed, the dioxobilane **1** appears to be formed stereoselectively, as no evidence for an isomer of it is available in senescent leaves of Norway maple. In an analytical HPLC study the amount of the catabolite **1** was found to correspond to nearly 50 % of the degraded chlorophyll. In contrast to the two *Hv*-UCCs, which were suggested to be formed by an oxidative transformation of *Hv*-NCC-1 within the plant cell,[10] the stereochemically aberrant dioxobilane **1** must have a different origin. The deduced *S* configuration at C-15 excludes a (rational) formation from known tetrapyrroles with structures like those found in *Hv*-UCC-1 and *Hv*-NCC-1. Thus the stereochemical differences between the dioxobilane **1**, on one side, and *Hv*-NCC-1 and the stereo-unselectively formed, epimeric *Hv*-UCCs, on the other, imply divergent paths for their formation.

The absence of a plausible path to the catabolite **1** from a natural NCC raises the question of its origin and at what point the pathway of the formation of **1** deviates from that of the other colorless chlorophyll catabolites. In particular, the crucial loss of the formyl group at C-6 must precede the acid-induced isomerization of an FCC to a corresponding NCC, which determines the *R* configuration at C-15.[20] An oxidative deformylation of a corresponding FCC is thus proposed as the most likely divergent step in the formation of **1** (see Scheme 2). It implies the formation of a previously unobserved and presumably short-lived tetrapyrrole of the type of 6-*nor-*FCC **2**, followed by a stereoselective isomerization of the latter to provide the dioxobilane **1** with *S* configuration at C-15 (see Scheme 2). The implied isomerization would thus lead to an effective diastereoselectivity opposite to that of the acid-induced isomerization of FCCs to NCCs. Further studies are required to assess the relevance of this catabolic path and of the intermediacy of a catabolite, such as **2** (see Scheme 2). Indeed, in senescent *Arabidopsis thaliana* leaves,[23] we have observed a fluorescent tetrapyrrole that exhibits the UV absorption properties expected for the chromophore of the hypothetical 6-*nor*-FCC **2**.[24]

The early steps of chlorophyll breakdown in higher plants occur in senescent chloroplasts and provide the “primary” FCCs by a common path.[4] However, at the subsequent stage of “secondary” FCCs (*s*FCCs) a range of (enzyme-catalyzed) side-chain modifications were revealed recently; these reactions are proposed to occur in the cytosol of the senescent plant cell[4] and give “modified” and “hypermodified” FCCs (*hm*FCCs) in some senescent leaves[26, 27] (e.g. of *Spathiphyllum wallisii*),[27] and in ripening bananas[28, 29] (see Scheme 2). The dioxobilane **1** suggests another type of a natural transformation of a catabolite in a higher plant, now involving deformylation at C-6. The hypothetical 6-*nor*-FCC **2** would be the first fluorescent chlorophyll catabolite that reflects a natural degradation of the basic tetrapyrrole skeleton of an FCC (without affecting its fluorophore moiety). Thus, the discovery of the dioxobilane **1** as the clearly major tetrapyrrolic chlorophyll catabolite in senescent leaves of Norway maple indicates a path of chlorophyll breakdown that diverges from that previously found in other deciduous trees. Indeed, Norway maple appears to be the first representative of the plant family of the *Sapindáceae*,[30] in which chlorophyll catabolites have been characterized.

Unlike the widely occurring NCCs and most other known natural chlorophyll catabolites,[7, 8] the colorless linear tetrapyrrole **1** lacks the formyl group at the cleavage site of the porphyrinoid macrocycle. Its structure is reminiscent of bile pigments, such as bilirubine and the phytobilins,[16] which are products of heme breakdown, and which are important constituents of the metabolisms in mammals and plants.[31] The suggested directed breakdown of chlorophyll to dioxobilanes, such as **1**, may thus be a further sign of the biological relevance of such chlorophyll-derived linear tetrapyrroles in plants. Indeed, chlorophyll breakdown has primarily been considered as a detoxification process and NCCs as mere detoxification products.[9] However, consideration of a further physiological role of tetrapyrrolic chlorophyll catabolites was suggested, when NCCs were revealed to be effective antioxidants in ripening fruit.[32] The deduced enzyme-catalyzed formation of blue-luminescent and “persistent” hypermodified FCCs, as well as the divergent paths of chlorophyll breakdown outlined here, all clearly strengthen the alternative view that the physiological relevance of chlorophyll catabolites in higher plants—although still unknown—should not be discounted.

## Experimental Section

HPLC analysis: A senescent leaf of Norway maple was ground in a mortar with 0.5 g of sea sand and 3 mL of methanol (MeOH). A 200 μL portion of the extract was mixed with 800 μL of 50 mm phosphate puffer (pH 7). After centrifugation at 13 000 rpm for 5 min a 20 μL aliquot was analyzed by reversed-phase (RP) HPLC (see Figure 1); for experimental details see the Supporting Information.

Isolation of the catabolite **1** (see the Supporting Information for details): A 130 g sample of leaves of Norway maple (*Acer platanoídes*, see Figures 1 and S2 in the Supporting Information,), which were collected at early senescence on October 20, 2008 in the Hofgarten (Innsbruck), was freeze-dried and extracted with MeOH. The catabolite was enriched by precipitation from diethyl ether followed by MPLC. The raw product was purified by preparative HPLC (isocratic solvent composition; 50 mm potassium phosphate (pH 7.0)/MeOH 70:30). After “desalting” an analytically pure sample of **1** (4.8 mg) was obtained as a pale yellow solid.

Spectroscopy: See the Supporting Information for details. Characterization of catabolite **1**: UV/Vis (Hitachi U-3000, MeOH, *c*=2.25×10^−4^ m, *λ*_max_/nm (log *ɛ*)): 210 (4.75), 237 sh (4.49), 274 sh (4.00); CD (Jasco J715, MeOH, *c*=2.25×10^−4^ m): *λ*_max_/nm (Δɛ)=227 (−40), 254 sh (6), 283 (39), 314(−7) (see Figure 2). ^1^H and ^13^C NMR (see the Supporting Information). ESI-HRMS (Bruker FT-ICR Apex Ultra, ESI positive-ion mode, polypropylene glycol as internal mass standard, MeOH/water as solvent): *m*/*z*=705.2532 ([*M*+K]^+^; *m*/*z*_calc_[C_34_H_42_N_4_O_10_K_1_]^+^=705.2533).
