# Supplementary material for: A Dioxobilane as Product of a Divergent Path of Chlorophyll Breakdown in Norway Maple
Source: Angew Chem Int Ed Engl. 2011 Sep 16;50(45):10724–7. doi: 10.1002/anie.201103934 (PMC3262146; doi:10.1002/anie.201103934)
Supplement: Supplementary file 1 [file anie0050-10724-SD1.pdf]

Angewandte Chemie
Eine Zeitschrift der Gesellschaft Deutscher Chemiker

Supporting Information

© Wiley-VCH 2011

69451 Weinheim, Germany

A Dioxobilane as Product of a Divergent Path of Chlorophyll Breakdown in Norway Maple**

*Thomas Müller, Martina Rafelsberger, Clemens Vergeiner, and Bernhard Kräutler**

anie_201103934_sm_miscellaneous_information.pdf

Supporting Information

Materials.
Commercially available solvents (reagent-grade) were redistilled before use for extractions. HPLC grade methanol (MeOH) and diethyl ether was from Merck (Darmstadt, Germany) and Acros Organics (Geel, Belgium). Potassium dihydrogen phosphate puriss. p.a. and potassium phosphate dibasic-anhydrous puriss. p.a. were from Fluka (Buchs, Switzerland). 5g and 1 g Sep-Pak-C18 Cartridges were from Waters Associates. The pH values were measured with a WTW Sentix 21 electrode connected to a WTW pH535 digital pH meter.

Methods.
^{1}H- and ^{13}C-NMR spectroscopy: Bruker UltraShield 600 MHz or Varian UNITYplus 500 MHz spectrometer. δ in ppm with δ-(CHD_2OD)=3.39 ppm; J_{HH} (Hz); ^{13}C- signal assignment from heteronuclear *^{1}H- and ^{13}C-* HSQC & HMBC experiments. *Mass spectrometry:* Finnigan MAT 95-S, positive-ion mode, m/z (rel. abundance); 'Electrospray Ionization' (ESI): infusion, spray voltage 1.4 kV, solvent water/MeOH 1:1 (v/v). *HPLC:* Dionex Summit HPLC system with manual sampler, P680 pump, online degasser and diode array detector. Injection loop 1 ml (Rheodyne injection valve). Data were collected and processed with Chromeleon V6.50. a) *Analytical HPLC:* Phenomenex HyperClone ODS 5μm 250 x 4.6mm i.d. column at 20°C protected with a Phenomenex ODS 4 mm x 3 mm i.d. precolumn was used with a flow rate 0.5 $ml.min^{-1}$. Solvent A: 50 mM potassium phosphate (pH 7.0), solvent B: MeOH; standard solvent compositions: (A/B) as function of time (0 - 120 min): 0 - 10: 80/20; 10 - 70: 80/20 to 40/60; 70 - 90: 40/60; 90 - 95: 40/60 to 0/100; 95 - 115: 0/100; 115 - 120: 0/100 to 80/20. b) *Preparative HPLC:* Phenomenex HyperClone ODS 5μm 250 x 21.2mm i.d. column at 20°C protected with a Phenomenex ODS 10 mm x 5 mm i.d. precolumn was used with a flow rate 5 $ml.min^{-1}$. Solvent A: 50 mM potassium phosphate (pH 7.0), solvent B: MeOH; isocratic solvent compositions: (A/B): 70/30. *MPLC*: Büchi MPLC system equipped with two C-605 pumps, a C-615 pump manager and a C-630 UV-detector at 254 nm. A 460 x 55 mm i.d. column at 20°C filled with Phenomenex Spepra-C18-E, 50μm, 65A was used with a flow rate of 10 $ml.min^{-1}$. Solvent A: 50 mM potassium phosphate (pH 7.0), solvent B: MeOH; isocratic solvent compositions: (A/B): 75/25.

Isolation of Catabolite 1.
Senescent *Acer platanoídes* leaves were collected on October 20th, 2008 and were (freshly) frozen in liquid nitrogen and stored at -80°C. 130g of the leaves were freeze-dried in vacuo until a dry-weight of about 48 g (reached within 4 days). The dried leaves

–

were pulverized and extracted with 120 ml of MeOH. The suspension was filtrated with suction over a Buchner funnel. The remaining filter cake was thoroughly washed with two portions of 30 ml of MeOH. The combined methanolic extracts were added portion wise to 315 ml of diethyl ether to precipitate a chlorophyll catabolite enriched raw product. After filtration with suction the light-brown precipitate was dried *in vacuo* (dry-weight: 1.39 g) and stored at -80°C for further purification.
For further purification by MPLC the raw product was re-dissolved in 70 ml of potassium phosphate buffer solution (50 mM; pH = 7). After centrifugation at 5000 rpm for 10 min a clear red-brownish solution was injected into the MPLC system. Under isocratic conditions a fraction containing catabolite **1** could be collected after 35 min. The collected fraction was concentrated to 2/3 of its volume using a rotary evaporator at r.t. For de-salting, the aqueous solution was then applied to a pre-conditioned Sepak-cartridge, washed with 40 ml of water and eluted with a minimum amount of MeOH. After the evaporation of the solvents *in vacuo* 88 mg of 'highly enriched' catabolite 1 could be obtained. Analytically pure samples for spectral characterization were obtained after further preparative HPLC separation: 20 mg of the 'highly enriched' fraction were dissolved in 1 ml of potasium phosphate buffer (pH7). After centrifugation at 13000 rpm for 10 min a clear light-brownish solution was injected into the preparative HPLC system. Under isocratic conditions a fraction containing catabolite **1** could be collected after 35 min. The collected fraction was concentrated to 2/3 of its volume using a rotary evaporator at r.t. For de-salting, the aqueous solution was then applied to a pre-conditioned Sepak-cartridge, washed with 20 ml of water and eluted with a minimum amount of MeOH. After the evaporation of the solvents *in vacuo* 4.8 mg of catabolite **1** could be isolated.

Spectroanalytical Data of catabolite 1.
HPLC: Retention time in analytical HPLC (rt) = 35 min, see main text and Figure 1).
UV/Vis (c = 2.25*10^{-4}M, MeOH): λ_{max} (log ε) = 210 (4.75), 237sh (4.49), 274sh (4.00).
CD(c=2.25*10^{-4}M, MeOH): λ_{max}/nm (Δε) = 227 (-40), 254sh (6), 283 (39), 314(-7).
^{1}H-NMR (500 MHz, CD_3OD, see Figure S5): δ [ppm] = 1.74 (s, 3H, H_3C-7^1); 1.97 (s, 3H, H_3C-18^1); 2.02 (s, 3H, H_3C-2^1); 2.09 (s, 3H, H_3C-12^1); 2.37 (m, 2H, H_2C-17^2); 2.49 (m, 1H, H_AC-8^1); 2.55 (dd, 1H, J = 8.6/14.6 Hz, H_AC-20); 2.63 (m, 1H, H_AC-10); 2.67 (m, 1H, H_AC-17^1); 2.75 (m, 1H, H_BC-8^1); 2.78 (m, 1H, H_BC-17^1); 2.89 (dd, 1H, J = 4.6/14.7 Hz, H_BC-20); 3.04 (dd, 1H, J = 4.6/14.8 Hz, H_BC-10); 3.61 (m, 1H, HC-3^2); 3.67 (m, 2H, H_2C-8^2); 3.74 (s, 3H, H_3C-13^5); 4.12 (dd, 1H, J = 4.6/8.5 Hz, HC-1); 4.40 (m, 1H, J = 5/11 Hz, HC-9); 4.54 (1H, HC-3^1)); 4.90 (m, 1H, HC-15). ^{13}C-NMR (125 MHz, CD_3OD, ^{13}C- signal assignment from HSQC & HMBC experiments): δ = 8.1 (7^1); 9.0 (18^1); 9.0 (12^1); 12.3 (2^1); 22.1 (17^1); 29.5 (20); 29.5 (10); 30.5 (8^1); 52.8 (13^5); 60.3 (9); 61.2 (8^2); 62.1 (1); 65.9 (3^2); 68.7 (3^1); 112.4 (12); 115.6 (18); 121.1 (17); 124.3 (16); 124.3 (19); 125.4 (13); 130.9 (3); 130.9 (7); 134.1 (11); 155.9 (8); 159.2 (2); 171.1 (13^3); 175.7 (4); 176.5 (6); 182.0 (17^3); 191.8 (13^1).

^{1}H-NMR (600 MHz, H_2O:D_2O=9:1 (v/v), 283K): δ (in ppm) = 1.54 (s, 3H, H_3C-7^1), 1.81 (s, 3H, H_3C-18^1), 1.93 (s, 3H, H_3C-2^1), 1.94 (s, 3H, H_3C-12^1), 2.12-2.25 (m, 2H, H_2C-17^2), 2.39 (m, 1H, H_AC-8^1), 2.48 (m, 2H, H_2C-17^1), 2.64 (m, 1H, H_BC-8^1), 2.66 (m, 1H, H_AC-10), 2.68 (m, 1H, H_AC-20), 2.84 (dd, 1H, J = 4.4/15.5 Hz, H_BC-20), 2.98 (dd, 1H, J = 4.3/16.3 Hz, H_BC-10), 3.35 (dd, 1H, J = 4.3/11.7 Hz, H_AC-3^2), 3.47 (m, 1H, J= ca. 7.9/11.7 Hz; H_BC-3^2), 3.56-3.65 (m, 2H, H_2C-8^2), 3.67 (s, 3H, H_3C-13^5), 3.70 (d, 1H, J = 2.0 Hz, HC-13^2), 4.12 (t, 1H, J = 5.0 Hz, HC-1), 4.30 (m, 1H, J = ca. 5.1 Hz, HC-9), 4.44 (dd, 1H, J = 4.9/7.8 Hz, HC-

3^1), 4.73 (HC-15, superimposed by the water signal), 7.77 (s, 1H, HN-21), 8.06 (s, 1H, HN-22), 9.33 (s, 1H, HN-24), 10.65 (s, 1H, HN-23).

MS (ESI): m/z (%) = 711.14 ($[M-H+2Na]^+$;10), 705.19 ($[M-K]^+$;25), 689.19 ($[M+Na]^+$;50), 667.19 ($[M+H]^+$;100), 635.11 ($[M+H-MeOH]^+$;12), 510.11 ($[M+H-RingA]^+$;18).
MS (ESI; CID 40V): m/z (%) = 711.14 ($[M-H+2Na]^+$;38), 705.19 ($[M+K]^+$;67), 689.19 ($[M+Na]^+$;100), 667.19 ($[M+H]^+$;15), 635.11 ($[M+H-MeOH]^+$;57), 617.17 (8), 510.11 ($[M+H-RingA]^+$;77), 496.61 (7), 478.06 (25), 369.04 (15), 339.06 (22), 278.93 (24).

Determination of chlorophyll and catabolite 1 in green and senescent *Acer platanoídes* leaves by UV/Vis spectroscopy and HPLC.
15-70 cm^2 of a *Acer platanoides* leaf (area was determined by photographical integration) was ground in a mortar with 0.5 g sea sand and 10 ml of MeOH. The slurry was filtered through a sintered glass filter, and the residue was ground in a mortar with 10 ml of MeOH. The procedure was repeated until the residue was colorless. The combined methanolic extracts were diluted with MeOH to an exact volume of 50.00 ml. 1.000 ml of the leaf extract was further diluted 1:1 (v/v) with MeOH and analyzed by UV/Vis spectroscopy. For analytical HPLC 600 µl of the methanolic leaf extract were diluted with 2.400 ml of 50mM phosphate puffer solution (pH=7). After centrifugation at 1300 rpm for 5 min 2.000 ml of the clear supernatant was injected to analytical HPLC (for the gradient see supporting section 'Methods'). For quantification of the catabolite **1** a standard was prepared by dissolving catabolite **1** (c = 0.37 x 10^{-8} mol.l–1) in 50 mM potassium phosphate (pH 7.0)/MeOH 80:20 (v/v).
UV/Vis quantification of the chlorophylls: Data analysis is based on [S1]. Analysis of green Norway Maple leaves (see Fig. S1, left side): 38.19 ± 1.36 $µg.cm^{-2}$ chlorophyll a and b were found. Analysis of senescent Norway maple leaves (see Fig. S1, right side): 8.73 ± 0.58 $µg.cm^{-2}$ chlorophyll a and b were found.
HPLC quantification of catabolite **1**: Analysis of green Norway maple leaves (see Fig. S1, left side): catabolite **1** could not be detected. Analysis of senescent Norway Maple leaves (see Fig. S1, right side): 10.31 ± 0.52 $µg.cm^{-2}$ of catabolite **1** were found. This corresponds to a 46.9% recovery of the chlorophylls degraded at the investigated stage of senescence (29.46 $µg.cm^{-2}$; see UV/Vis quantification of the chlorophylls and Figure S1)

CD spectroscopy of *Hv*-UCCs from senescent leaves of barley (*Hordeum vulgare*).
The yellow areas of six senescent barley leaves were ground in a mortar with 0.5 g sea sand and 3 ml of MeOH. The suspension was centrifuged at 13000 rpm for 2 min. The methanolic supernatant was concentrated with a rotary evaporator to about 300µl and was then diluted with 1200µl of 50 mM potassium phosphate buffer at pH = 7.0. After centrifugation at 13000 rpm for 10 min, 1.4 ml of the clear supernatant was injected into analytical HPLC. Solvent A: 50 mM potassium phosphate (pH 7.0), solvent B: MeOH; isocratic solvent compositions: (A/B): 75/25. Two UCC fractions at 32,4 min and 46,1 min (around 400µl each) were collected and analyzed by UV/Vis and CD spectroscopy with a mixture of 50 mM potassium phosphate buffer at pH = 7.0 and MeOH (v/v = 75:25) as reference.

Co-injection experiments using analytical HPLC.
Analytical HPLC was used to analyze freshly prepared extracts of senescent Norway Maple and Barley leaves. Fractions of both, *Acer platanoídes* catabolite **1** as well as *Hv*-UCC-1 eluted at a retention time of about 35 min (Figure S6, Panel A and B). For Co-

injection experiments, isolated catabolite **1** (Figure S5, Panel C) and a 1:1 mixture of catabolite **1** and *Hv*-UCC-1 (Figure S6, Panel C) were analyzed by analytical HPLC. Solvent A: 50 mM potassium phosphate (pH 7.0), solvent B: MeOH; isocratic solvent compositions: (A/B): 65/35. Catabolite **1** and *Hv*-UCC-1 co-eluted at 18.7 min, their naturally occurring epimers at 15,8 min (Figure S6, Panel C and D).

References.

[S1] Porra., R. J., Thompson, W. A., Kriedemann, P. E. Biochim. Biophys. Acta **1989**, 975, 384.
[S2] F. G. Losey, N. Engel, *J. Biol. Chem.* **2001**, *276*, 8643.

Supporting Table and Figures

Supporting Table 1. Signal assignments for 600-MHz ^1H-NMR spectra (in $H_2O:D_2O=9:1$ (v/v), 283 K, δ in ppm) of catabolite **1** from *Acer platanoídes* and *Hv*-UCC-1 from *Hordeum vulgare* [S2].

Signal Assignment	δH (1)	δH (*Hv*-UCC-1)	δH(1) - δH(*Hv*-UCC-1)
$H_3C\text{-}7^1$	1.54	1.54	0.00
$H_3C\text{-}18^1$	1.81	1.82	-0.01
$H_3C\text{-}2^1$	1.93	1.91	0.02
$H_3C\text{-}12^1$	1.94	1.97	-0.03
$H_2C\text{-}17^2$	2.12-2.25	2.10-2.20	0.02 - 0.05
$H_AC\text{-}8^1$	2.39	2.40	-0.01
$H_2C\text{-}17^1$	2.48	2.45-2.50	0.03 – (-0.02)
$H_BC\text{-}8^1$	2.64	2.64	0.00
$H_AC\text{-}10$	2.66	2.68-2.74	(-0.02) - (-0.08)
$H_AC\text{-}20$	2.68	2.68-2-74	0.00 - (-0.06)
$H_BC\text{-}20$	2.84	2.85	-0.01
$H_BC\text{-}10$	2.98	3.01	-0.03
$H_AC\text{-}3^2$	3.35 [a)]	3.34 [b)]	0.01
$H_BC\text{-}3^2$	3.47	3.56-3.66	(-0.09) - (-0.19)
$H_2C\text{-}8^2$	3.56-3.65	3.56-3.66	0.00 - (-0.01)
$H_3C\text{-}13^5$	3.67	3.69	-0.02
$HC\text{-}13^2$	3.70		
HC-1	4.12	4.16	-0.04
HC-9	4.30	4.32	-0.02
$HC\text{-}3^1$	4.44	4.44	0.00
HC-15	4.73	4.74	-0.01
HN-21	7.77		
HN-22	8.06		
HN-24	9.33		
HN-23	10.65		

[a)] J_{HH} = 4.6Hz/7.9Hz/11.7Hz [b)] J_{HH} = 4.3Hz/4.6Hz/11.7Hz

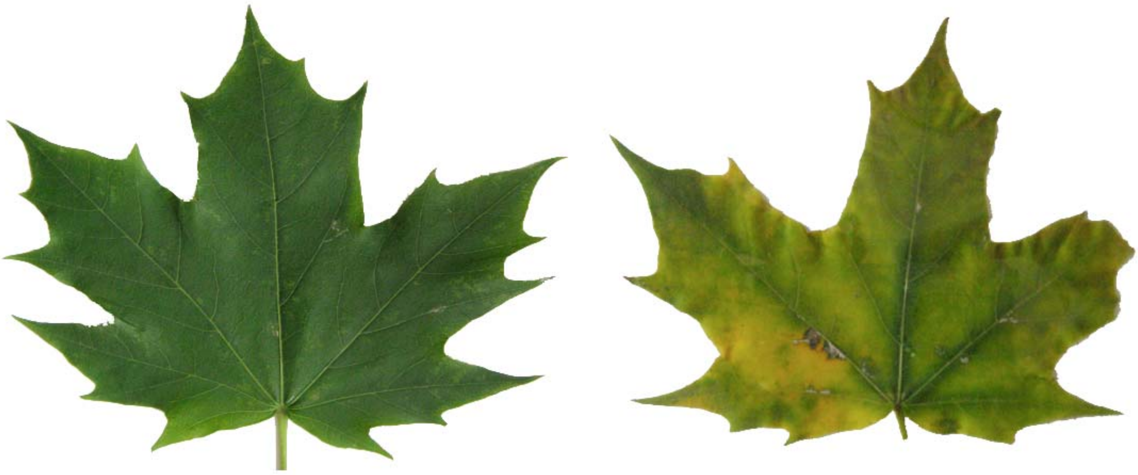

Supporting Figure S1: Green and early senescent *Acer platanoídes* leaves collected at the Hofgarten in Innsbruck on October 2nd, 2008 (left) and October 20th, 2008 (right).

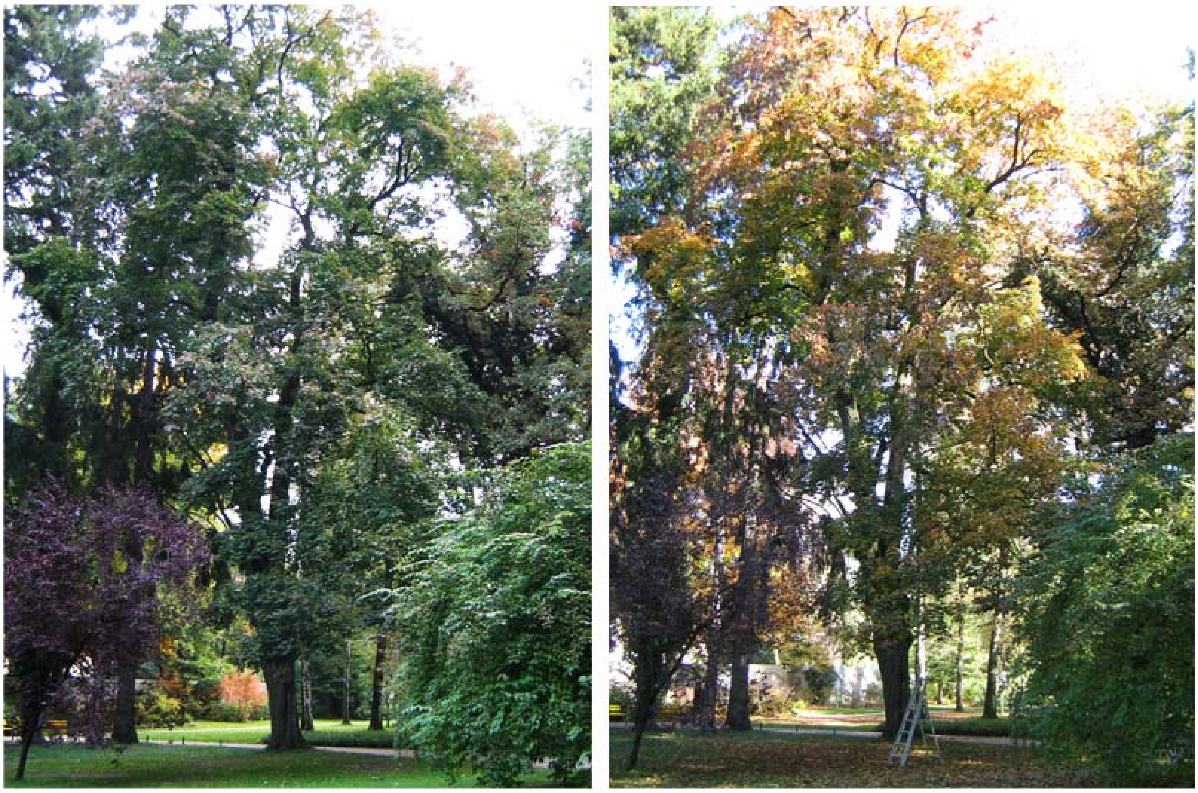

Supporting Figure S2: *Acer platanoídes* tree at the Hofgarten in Innsbruck. Pictures were taken on October 2nd, 2008 (left) and October 20th, 2008 (right)

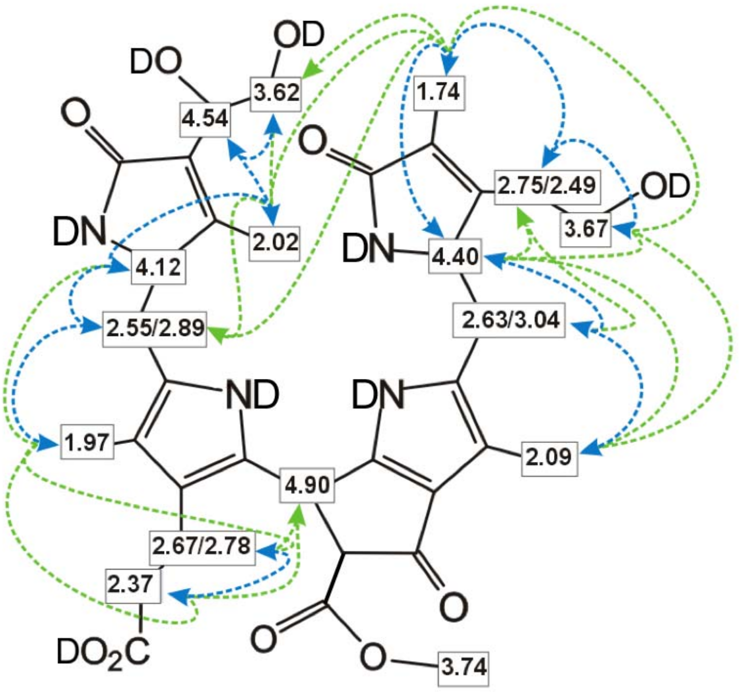

Supporting Figure S3: Graphical representations of the ^{1}H-NMR chemical shift data of catabolite **1** in CD_3OD: signal assignments of C-bound H-atoms (blue arrows represent homonuclear $^{1}H,^{1}H$-COSY correlations; green arrows indicate additional homonuclear $^{1}H,^{1}H$-ROESY correlations)

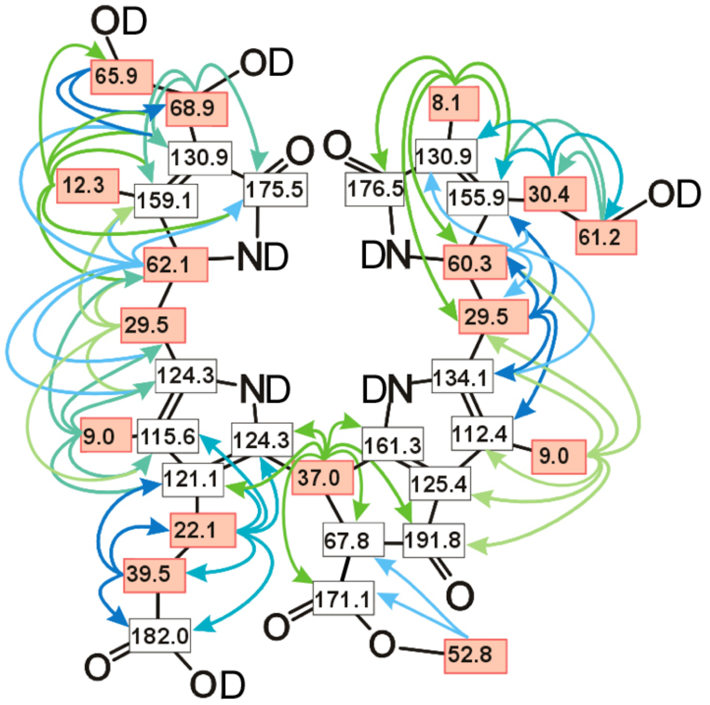

Supporting Figure S4: Graphical representations of the heteronuclear $^{1}H,^{13}C$-HSQC and $^{1}H,^{13}C$-HBMC chemical shift data of catabolite **1** in CD_3OD: H-bound C-atoms assigned from the $^{1}H,^{13}C$-HSQC experiment are marked with coloured boxes, C-atoms assigned from the $^{1}H,^{13}C$-HMBC experiment are marked with white boxes. All coloured arrows indicate heteronuclear $^{1}H,^{13}C$-HMBC correlations.

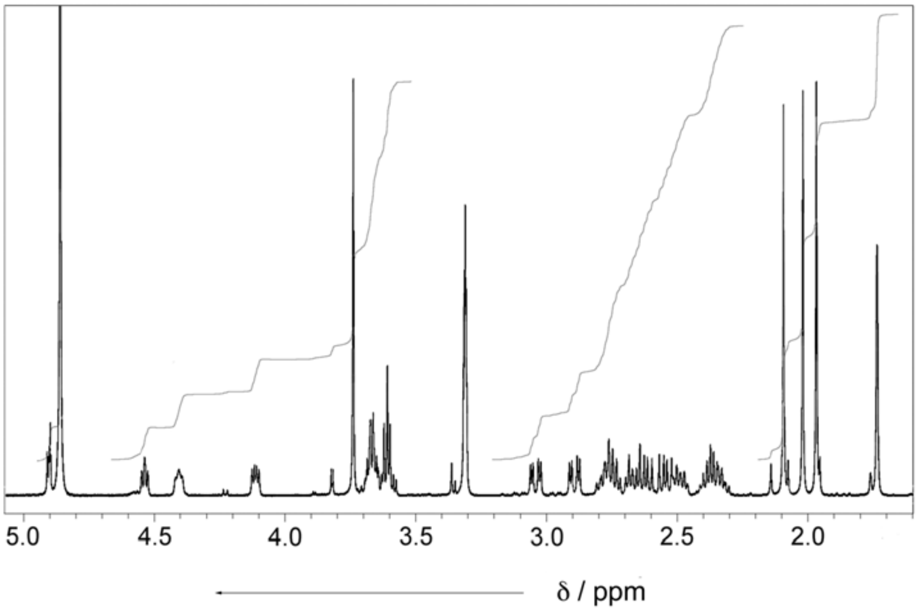

Supporting Figure S5. 500-MHz ^{1}H-NMR-spectrum of the catabolite **1** (in CD_3OD)

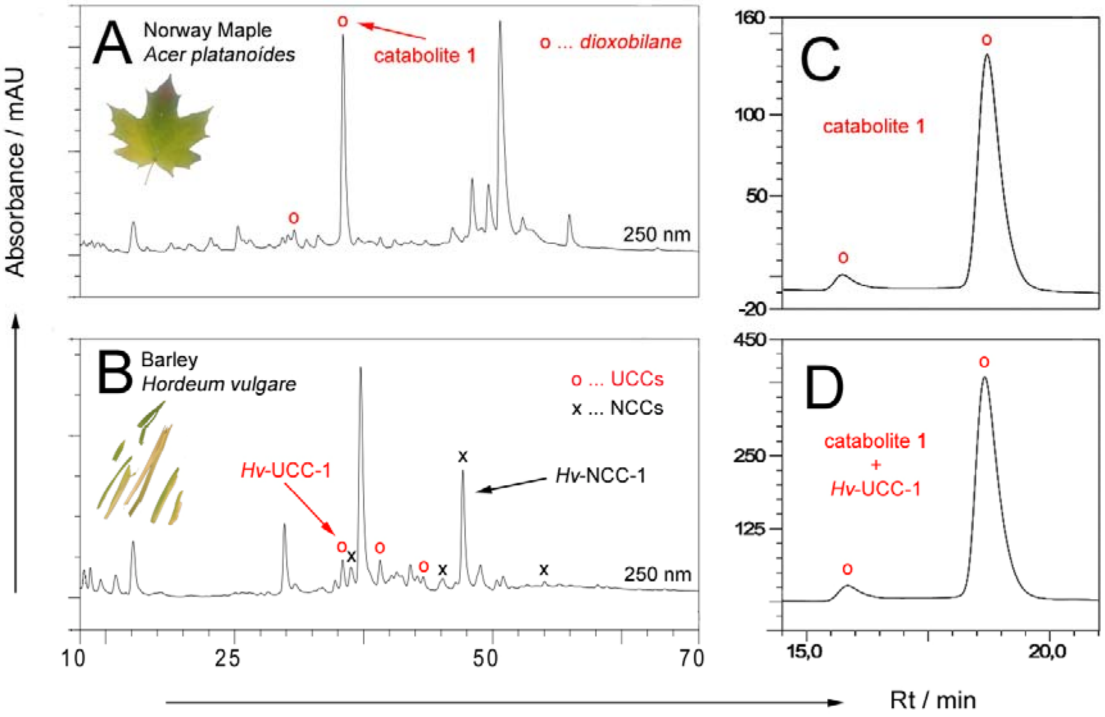

Supporting Figure S6. Co-injection experiments using analytical HPLC. Panels A and B: HPL-chromatograms of methanolic extracts from senescent leaves from Norway Maple and barley (250 nm traces; dioxobilanes and urobilinogenoidic chlorophyll catabolites (UCCs) are marked red circles, nonfluorescent chlorophyll catabolites (NCCs) are marked with black crosses. Panels C and D: Co-injection of catabolite **1** from *Acer platanoídes* and *Hv*-UCC-1 from *Hordeum vulgare*.
